# Children with Rare Nager Syndrome—Literature Review, Clinical and Physiotherapeutic Management

**DOI:** 10.3390/genes15010029

**Published:** 2023-12-24

**Authors:** Bożena Anna Marszałek-Kruk, Andrzej Myśliwiec, Anna Lipowicz, Wojciech Wolański, Małgorzata Kulesa-Mrowiecka, Krzysztof Dowgierd

**Affiliations:** 1 Department of Genetics, Wroclaw University of Environmental and Life Sciences, 51-631 Wroclaw, Poland; 2 Laboratory of Physiotherapy and Physioprevention, Institute of Physiotherapy and Health Sciences, Academy of Physical Education in Katowice, 40-065 Katowice, Poland; 3 Department of Anthropology, Faculty of Biology and Animal Science, Wroclaw University of Environmental and Life Sciences, 50-375 Wroclaw, Poland; 4 Department of Biomechatronics, Faculty of Biomedical Engineering, Silesian University of Technology, 41-800 Zabrze, Poland; 5 Department of Rehabilitation in Internal Diseases, Faculty of Health Sciences, Jagiellonian University Medical College, 31-126 Krakow, Poland; 6 Head and Neck Surgery Clinic for Children and Young Adults, Department of Clinical Pediatrics, Collegium Medicum, University of Warmia and Mazury, 10-561 Olsztyn, Poland

**Keywords:** Nager syndrome, *SF3B4* gene, acrofacial dysostoses, clinical treatment

## Abstract

Nager syndrome is a rare human developmental disorder characterized by craniofacial defects including the downward slanting of the palpebral fissures, cleft palate, limb deformities, mandibular hypoplasia, hypoplasia or absence of thumbs, microretrognathia, and ankylosis of the temporomandibular joint. The prevalence is very rare and the literature describes only about a hundred cases of Nager syndrome. There is evidence of autosomal dominant and autosomal recessive inheritance for Nager syndrome, suggesting genetic heterogeneity. The majority of the described causes of Nager syndrome include pathogenic variants in the *SF3B4* gene, which encodes a component of the spliceosome; therefore, the syndrome belongs to the spliceosomopathy group of diseases. The diagnosis is made on the basis of physical and radiological examination and detection of mutations in the *SF3B4* gene. Due to the diversity of defects associated with Nager syndrome, patients require multidisciplinary, complex, and long-lasting treatment. Usually, it starts from birth until the age of twenty years. The surgical procedures vary over a patient’s lifetime and are related to the needed function. First, breathing and feeding must be facilitated; then, oral and facial clefts should be addressed, followed by correcting eyelid deformities and cheekbone reconstruction. In later age, a surgery of the nose and external ear is performed. Speech and hearing disorders require specialized logopedic treatment. A defect of the thumb is treated by transplanting a tendon and muscle or transferring the position of the index finger. In addition to surgery, in order to maximize a patient’s benefit and to reduce functional insufficiency, complementary treatments such as rehabilitation and physiotherapy are recommended. In our study, we describe eight patients of different ages with various cases of Nager syndrome. The aim of our work was to present the actual genetic knowledge on this disease and its treatment procedures.

## 1. Background

Nager syndrome (NS) (OMIM 154400; ORPHA 245), known as Nager acrofacial dysostosis and limb–facial dysostosis AFD1, is a rare human developmental disorder characterized by craniofacial defects including downward slanting of the palpebral fissures, micrognathia, cleft palate, limb deformities, mandibular hypoplasia, hypoplasia or absence of thumbs, absent lower medial eyelashes, mandibular hypoplasia, microretrognathia, and ankylosis of the temporomandibular joint (TMJ) [1]. In patients with Nager syndrome, TMJ may arise after mandibular distraction osteogenesis [2]. Severe micrognathia results in the need for tracheostomy- and gastrostomy-tube placement [3].

Nager acrofacial dysostosis was first described in 1948 by Nager and de Reyenie. Nager syndrome occurs in the general population at a frequency of 3 per 1,000,000 live births. The incidence of the disease is slightly higher in females than in male individuals. Most cases are sporadic and are not related to other family members. Due to the similarity of the present congenital defects, Nager syndrome is differentiated from Goldenhar, Treacher Collins, Pierre Robin, and Genée–Wiedemann syndromes [4]. Only in Nager syndrome limb anomalies are observed (i.a., absence of thumbs, clinodactyly (curvature of the fingers of the hand), syndactylia (fusion of the fingers), shortening of the forearms due to the absence or partially formed radius bone, osteochondrosis of the radial-ulnar joint, and lack of upright movement at the elbow joint, which are the distinguishing criteria from other branchial arches’ syndromes) [5]. Nager syndrome is diagnosed based on the patient’s history, clinical signs, additional tests such as X-rays of the head and limbs, and genetic testing. The intensity of defects varies from poorly expressed to extensive defects of very significant severity.

The aim of this work was to present knowledge about the genetic basis of Nager syndrome. Our paper also presents proposed treatment procedures and methods of possible rehabilitation for Nager syndrome. The reason behind it is because the prognosis for survival after the neonatal–infant period is good and it is important to apply effective specialized surgical and physiotherapeutic therapy, personalized to individual needs, in order to improve the patient’s functioning.

## 2. Molecular Diagnosis

There is evidence of autosomal dominant and autosomal recessive inheritance for Nager syndrome, suggesting genetic heterogeneity [6]. No cases of intellectual disability are described. Up to now, in the literature, a clear correlation between the pathogenic variant and particular phenotype of patients had been missing. It has not been pointed out which pathogenic variant causes a particular feature of appearance; however, the newest articles suggest that a correlation may exist [7]. In the literature, studies have described approximately 100 cases of patients with Nager syndrome so far; only 10 of them have been indicated prenatally. Prenatal testing using an ultrasound revealed: suspected mandibular hypoplasia, where the mandible was relatively atrophic, facial anomalies, and limb anomalies [8].

The major causes of Nager syndrome are pathogenic variants in the Splicing Factor 3b Subunit 4 (*SF3B4*) gene, which encodes a component of the spliceosome. Therefore, Nager syndrome belongs to a group of diseases known as spliceosomopathies [9]. Bernier et al. (2012) described that a haploinsufficiency of *SF3B4* is responsible for more than 50% of clinically diagnosed patients [10].

The *SF3B4* gene (ID: 10262) consists of six exons and it is located on chromosome 1q21.2. The *SF3B4* gene encodes a SAP49 protein, which has 424 amino acids. This protein belongs to the mammalian SF3b complex and plays a role in RNA splicing [11]. It is involved in early embryogenesis and skeletal development [12]. The *SF3B4* gene is expressed in mouse embryos on the fore and hind limbs at an early stage of development, suggesting its involvement in skeletal development [13]. Nonsense, frameshift, and splice site mutations have been identified in this gene [10].

Byrd et al. (1988) described four patients with Nager acrofacial dysostosis, including one pair of monozygotic twins [14]. Opitz et al. (2000) described the first case of TMJ ankylosis correction with a total joint replacement for patients with Nager syndrome [15]. Ansart-Franquet et al. (2009) described cases of Nager syndrome with prenatal diagnosis at 22 weeks of gestation in a twin pregnancy [16]. A postmortem X-ray analysis confirmed retrognathia in both twins, partial radial-ulnar synostosis with bilateral thumb agenesis in twin 1, and bilateral radial-ulnar synostosis and thumb agenesis in twin 2. Both twins showed a proper male karyotype (46, XY). 

Lin (2012) described the first recorded case in Taiwan. It was a 3-year-old girl with the typical flat-nasal-bridge phenotype of Nager syndrome [17]. Bernier et al. (2012) described 18 heterozygous variants in the *SF3B4* gene that manifested as Nager syndrome [10]. Czeschik et al. (2013) identified heterozygous variants in seven patients [18]. Petit et al. (2014) reported 14 families, comprising 18 patients, with Nager syndrome and identified pathogenic variants in the *SF3B4* gene in 64% of these families; heterozygous loss-of-function mutations were identified in nine of these patients [19]. McPherson et al. (2014) presented a heterogeneous de novo mutation in the *SF3B4* gene in a patient with Rodriquez syndrome, which is considered a severe form of Nager syndrome [20].

Patients with Nager syndrome are characterized by symptoms similar to those of patients with Treacher Collins syndrome (TCS), which has been known to lead to misdiagnoses. Zhao and Yang (2020) described a case of a newborn with Nager syndrome, who was first diagnosed with TCS. They detected a c.1A>G substitution in the *SF3B4* gene [21].

Drendel et al. (2021) described the deletion of the *SF3B4* gene, spanning exons 3–6, in one person. This work describes the prenatal diagnosis of Nager syndrome via chromosomal microarray [22]. Tkemaladze et al. (2022) described the first case of Nager syndrome in Georgia [23].

Ulhaq et al. (2023) analyzed 24 articles involving 84 patients with Nager syndrome, including nine patients with Rodriquez syndrome. In total, 76% of the subjects had variants in the *SF3B4* gene. They identified 35 pathogenic variants in the *SF3B4* gene in exons 1 (c.1A>G) and 5 (c.1060dupC). They noted that patients with *SF3B4* frameshift variants had more severe clinical symptoms. Patients with variants in exons 2 (50%) and 3 (29.4%) showed a higher rate of cardiac defects. They demonstrated a possible genotype–phenotype relationship in Nager syndrome [7].

Genetic testing is crucial in the diagnosis of patients with Nager syndrome in order to exclude other diseases with similar clinical features. The aim of this work was to present genetic knowledge, treatment procedures, and methods of possible rehabilitation for Nager syndrome.

## 3. Material and Methods

**Figure 1:**
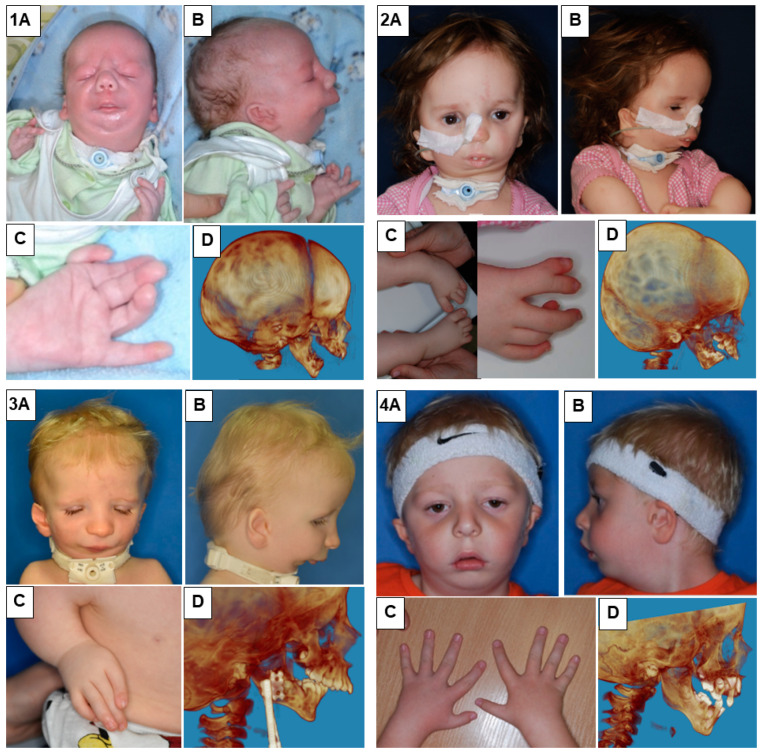
Patients with Nager syndrome: (1) newborn, (2) three years of age, (3) two years of age, and (4) five years of age. (**A**): front view. (**B**): lateral view. (**C**): (1) view of the hand with a vestigial undeveloped thumb, (2) the foot and lower leg with talipes equinovarus (equinus foot) and with abnormal toe structure, (3) the hand with a misaligned and undeveloped thumb as well as characteristic position of the arm due to contracture of the elbow joints and underdevelopment of the bones, and (4) patient with all five fingers on each hand. (**D**): 3D reconstruction of a CT image lateral projection.

**Figure 2:**
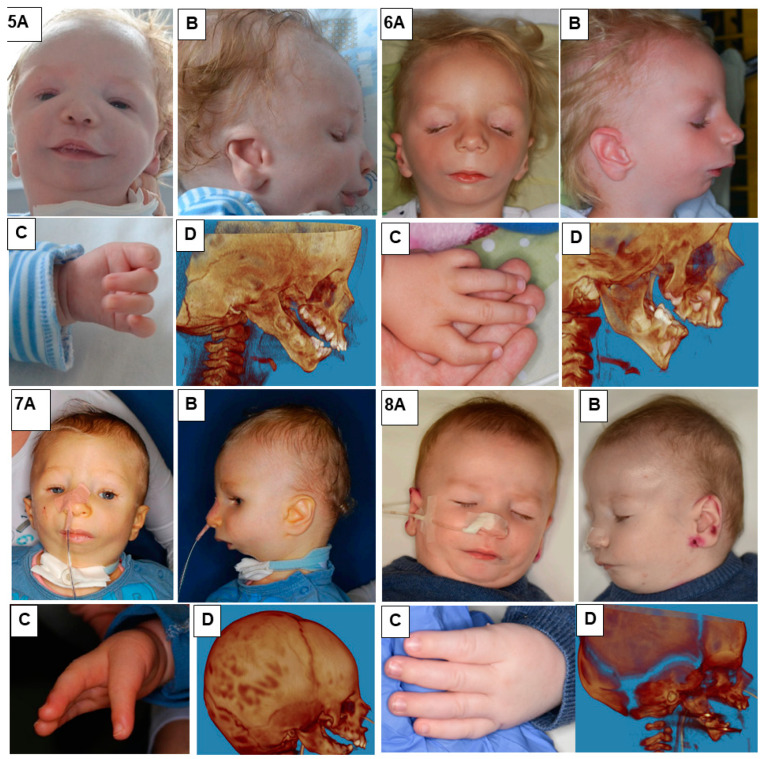
Patients with Nager syndrome: (5) newborn, (6) three years of age, (7) two years of age, and (8) two years of age. (**A**): front view. (**B**): lateral view. (**C**): (5) view of hand with residual undeveloped thumb, (6) hand with an incorrectly positioned and underdeveloped thumb, and (7,8) hand with congenital absence of a thumb. (**D**): 3D reconstruction of a CT image lateral projection from before treatment.

In our study, we presented eight patients of different ages with various cases of Nager syndrome. Next, clinical and physiotherapeutic approaches were described. The patients’ origin was Caucasian, from Central Europe, mostly from the Polish population. Table 1 presents phenotypes, conducted surgical procedures of the patients, as well as ages in years of the child depicted in the photograph, their sex, and the basis for the diagnosis of Nager syndrome. Photographs illustrating patients with Nager syndrome are shown in Figure 1 and Figure 2.

## 4. Clinical and Physiotherapeutic Management

Due to the diversity of defects associated with Nager syndrome, patients require multidisciplinary, complex, and long-lasting treatment. Therapy should be tailored to the specific needs of each individual. The treatment can start in the neonatal period and can be completed around 20 years of age. The surgical procedures change over time and are related to the needed function. First, breathing and feeding must be facilitated; then, oral and facial clefts; followed by eyelid deformity and cheekbone reconstruction. In later age, to improve one’s comfort of life, a surgery of the nose and external ear is performed. 

Nager syndrome is characterized by various dysfunctions in the area of the head and face as a result of the abnormal formation of the first and second branchial arches during prenatal development. In some individuals, the deformation of the limb buds is also observed. This implies that medical treatment ought to be carried out in specialized centers, where a multidisciplinary team approach can be applied. Such a multidisciplinary team should comprise at least a maxillofacial surgeon, laryngologist, hand surgeon, plastic surgeon, physiotherapist, audiologist, dentist and orthodontist, geneticist, and speech and language therapist, in addition to other pediatric specialists. The treatment of severe mandibular hypoplasia is performed via osteodistraction. Hearing devices are used to aid in the treatment of hearing loss. Speech and hearing disorders require specialized logopedic treatment. 

Due to the developmental changes in the impacted body parts, the increased body size, and the vital functions undertaken during patients’ growth, different medical practices are applied. The management of a newborn must focus on respiratory problems (tracheostomy) and feeding difficulties (gastrostomy). After birth, during the neonatal and infant period, the highest priority for clinicians is to stabilize the child’s breathing function via respiratory treatment. In some cases, a tracheostomy is necessary to allow the child to breathe. Some patients need support for breathing only at night. Feeding difficulties can be mitigated via enteral feeding, using a probe or gastrostomy directly into the stomach, bypassing the mouth and throat. These techniques allow children to breathe independently and make it possible to avoid tracheotomy. The procedure is quite often performed in the early stage of neonatal life. It is conducted in two steps in which the mandible is enlarged and its location is changed. Due to bone growth in early childhood and adolescence, another facial reconstruction surgery could be necessary.

Next, the surgical reconstruction of oral and facial clefts is performed. At a later age, an underdeveloped maxilla and mandible may require further treatment via osseous transplantation and/or mandibular distraction. CT (Computer Tomography) scans and 3D CAS (Computer Assisted Surgery) methods may supplement the diagnosis of rare syndromes and help in planning treatment and simulating procedures. Based on such support, preoperative planning allows for the design and application of customized medical implants. It is a long-term treatment, and in the case of most children, it has good results. At the same time, further surgical interventions of the mandible (orthognathic) and chin (genioplasty) can be conducted. Orthodontic treatment should be completed before mandibular surgery to help align the teeth in the correct orientation. The reconstruction of the midface, which comprises bone grafting or cheek implants, may be started at the age of about 16 years, when the growth processes of the skull are completed, earlier in girls and slightly later in boys. Depending on one’s individual needs and preferences, a surgery of the nose (rhinoplasty and/or septoplasty) may be needed to avoid airway obstructions.

Another phenotype of Nager syndrome is a defect of the thumb. If the thumb exists but is dysfunctional, a surgery to sustain the unstable joints and to reinforce the thumb can be conducted by taking a tendon or muscle from elsewhere in the hand. If the thumb is missing or unstable, the index finger can be transferred to the position of the thumb (pollicization).

Speech and hearing disorders require specialized logopedic treatment. Ears can be operated on even in late childhood by creating new ear replications based on the second ear or the parent’s ear if the patient is missing both ears. Ear reproduction only improves the appearance but does not restore one’s hearing function. Children with hearing impairment need the support of a hearing aid or cochlear implant as well as speech and language therapy.

Patients with pathologies common for Nager syndrome often require surgical correction of lower eyelid deformities, cheekbone reconstruction, fat grafting to increase facial volume, ear reconstruction, and orthognathic surgery to correct occlusion and their facial proportions. The age at which the procedures are performed depends on the severity of each deformity and how significantly it affects the child’s health.

Treatment is multidisciplinary and designed to meet the individual child’s needs and to maximize their results. The prognosis of treatment and the efficiency in Nager syndrome are variable and depend on the symptoms’ intensity, the severity of the defect, and its impact on basic functions such as breathing and hearing. The child’s health status must be monitored, especially during growth and adolescence. The surgical–medical processes ought to be supplemented via appropriate physiotherapy. The crucial role of physiotherapy is to ensure the functioning of the enhanced organs. After changing the structure, the patient must gain functional skills, e.g., chewing and breathing.

Functional improvement should include the activation of the mandible in all planes: tissue therapy of the masseter and temporal muscles and therapy of the suboccipital muscles and muscles of the neck. Techniques to support breathing by activating the chest and diaphragm are important elements [24]. Physiotherapy should start as early as possible to avoid functional deficits and should be carried out at least once a day [25]. The parents or guardians of the child ought to be instructed in basic nursing and activating techniques to ensure continuous improvement throughout the week. Moreover, rehabilitation should include the motor functions that have been disabled due to deformation or a lack of limb fragments. After a thorough functional and anatomical analysis, the child should be provided with harmonious development, taking into account all subsequent developmental stages, both those related to gross and fine motor skills [25]. In the case of ankylosis, after the joint release procedure, intensive rehabilitation should be performed from the first day after the procedure. After using analgesics, physiotherapy is recommended twice a day on an outpatient basis and every 2 h at home. From the first day after the procedure, jaw opening procedures should be performed after taking painkillers until the pain decreases to an acceptable level. Then, the improvement can be carried out without pharmacotherapy. Due to the presence of a postoperative wound, physiotherapy treatments had to be performed with sterile surgical gloves. Each time the treatment begins by opening the jaws and then wedges (e.g., silicone) are inserted between them. They are kept between the teeth for about 1 h. At home, mouth opening should be practiced and stabilizing wedges should be inserted to maintain the effect of the therapy for about 1 h. The therapy should begin with relaxing the internal and external tissues with a warm gel or massage [25].

The dominant techniques in physiotherapy of patients with face/head deformities are heat treatments and relaxing. They can be dosed in the form of passive heat or, in the absence of contraindications, generated heat; for example, that generated by Tecar or Indiba devices. Following relaxation, treatments are started to increase the range of mobility of jaw abduction as well as movements in other planes. As the child matures, active therapy is introduced, including exercises of the tongue and mimic muscles. In addition, exercises for the flexibility of the cervical spine, exercises for the muscles of the shoulder girdle, and active breathing exercises are advised. 

The physiotherapy procedures in this type of dysfunction were well described by Kulesa-Mrowiecka et al. (2021) [26]. The physiotherapy program should include manual therapy, myofascial release techniques, food- and drink-intake training, and sensory training. The main task of manual therapy in children with Nager syndrome is to eliminate contractures and to increase the mobility of the TMJ. After the operations, there are special physiotherapy procedures depending on the type of reconstruction. The first step in therapy should involve myofascial release techniques, fascia relaxation techniques, and transverse massage. All physiotherapy techniques are focused on the chewing muscles as well as on the suboccipital muscles, suprahyoid muscles, and infrahyoid muscles, which can be contracted due to congenital deformities. Joint mobilization can be performed using the extraoral grip with the stabilization of the child’s head. Mandible movement restriction initially prevents mobilization with the intraoral grips, which should be performed after achieving greater mouth opening. The articular slide should be moved in the opposite direction to the occurrence of pain (indirect mobilization) so that it is as comfortable as possible for the patient. The mobilization of the TMJ has to be performed in all directions of mandibular movement. During therapy, traction techniques for the TMJ can be also used, which positively influence the increase in mandibular movement. 

The next step involves passive manipulations with spatulas placed in the mouth to increase the jaw abduction movement (concave–convex techniques). Sensory hypersensitivity occurring in the orofacial area can block the achievement of natural functions (speech, drinking, and food eating). To eliminate this condition, the orofacial area should be massaged with different types of objects of various textures, such as a moistened glove, cotton swab, soft brush, and rough brush. Various types of fluids should also be used to reduce oral hypersensitivity in the following order of application: clear water, diluted apple juice, concentrated apple juice, diluted orange juice, and concentrated orange juice. This prepares the child for later food-intake training.

In food-intake training, it is important to improve all the stages of eating: gripping food with the lips, biting, chewing, and swallowing. The training should begin with feeding with a small teaspoon of liquid and soft foods, gradually passing through semi-liquid, crunchy products to hard products. The manner of drinking should also be trained in subsequent stages: a teaspoon, a cup, a tube, and a bottle; this required the activation of facial and masticatory muscles, especially the orbicularis oris muscle and the tongue and mandible protrusion movement, which were gradually achieved during therapy.

In the rehabilitation process, the role of parents and/or guardians is strongly highlighted. They should be given precise instructions for performing exercise programs at home. The parents should be given guidelines and a home exercise program for their child [27].

## 5. Conclusions

Nager syndrome is a very rare syndrome characterized by facial anomalies and limb defects. This disease is caused by abnormal differentiation during the development of the first and second branchial arches and limb buds. Diagnosis is based on genetic testing and a description of the clinical features. The phenotype of Nager syndrome is usually similar to the phenotypes of other rare dysmorphological syndromes: Treacher Collins syndrome (OMIM 154500), Miller syndrome (OMIN 263750, Postaxial acrofacial dysostosis), and Goldenhar syndrome (OMIM 164210). This makes it easy to misdiagnose.

The clinical diagnosis of Nager syndrome can also be confused with Rodriguez syndrome (OMIN 201170). Both syndromes are caused by pathogenic variants in the *SF3B4* gene and have similar phenotypic features; however, in Rodriguez syndrome, the defects are more severe and include lower-limb defects [28]. Some authors describe Rodriguez syndrome as a more severe case of Nager syndrome.

Approximately one third of studied Nager syndrome patients do not have pathogenic variants in the *SF3B4* gene [21]. In these cases, further diagnostics are required, preferably an analysis of the following genes: *TCOF1*, *POLR1D*, *POLR1C*, *POLR1B*, *DHODH*, and *EFTUD2*.

Finding each new mutation could improve the accuracy of this rare diagnosis. Nowadays, for the diagnosis of genetic diseases, Whole Exome Sequencing (WES) or even Whole Genome Sequencing (WGS) are used more and more. On the other hand, CT scans and X-ray methods may supplement the diagnosis of rare syndromes and help in planning treatment procedures. With the spread of 3D printing, it supports preoperative planning as well as being used for customized medical implants. It can be anticipated that, within a few years, as more cases are analyzed via WGS, it will be possible to show more correlations between the variants and the observed phenotypes.

Finally, due to the good prognosis, the main goal in the treatment of congenital malformations of different natures is to achieve functional, morphological, and cosmetic improvement. It is important to take an individual approach to the patient, adjusting the surgical and physiotherapeutic techniques used to improve the quality of life of the patient and his/her family.

## Figures and Tables

**Table 1:** Summarized phenotype characteristics and performed surgery operations of patients with Nager syndrome.

Patient	Age in Photos (in Years)	Sex	Diagnosis	Phenotype	Selected Surgeries Performed
1	newborn	M	*SF3B4* gene	- hypoplasia of the mandible - low-set auricles - bilateral conductive hearing loss - external ear canal atresia - undeveloped thumb	- tracheostomy - mandibular osteoplasty with implantation of distractors at age 1 - removal of mandibular distractions at age 1 - mandibular osteotomy with placement of distractors at age 2 - bilateral mandibular osteodistraction at age 5 - removal of mandibular distractors at age 5 - bilateral mandibular osteodistraction at age 7 - emergency tracheostomy for respiratory failure at age 7 - hearing implant
2	three	F	Clinical	- hypoplasia of the mandible - low-set auricles - low stature - bilateral congenital hearing loss of the conductive type - hypoplasia of the upper and lower limbs	- closure of submucosal secondary cleft palate at age 1 - tracheostomy - bilateral mandibular branch osteotomy with placement of distractors at age 2 - removal of mandibular distractors at age 2 - mandibular osteotomy with placement of distractors at age 3 - removal of mandibular distractors at age 4 - mandibular osteotomy procedure with placement of mandibular distractors at age 5 - gastroscopy with nutrient fistula creation and PEG placement at age 7 - bilateral mandibular osteotomy with placement of internal distractors at age 8 - removal of mandibular distractors at age 8
3	two	M	*SF3B4* gene	- hypoplasia of the mandible - low-set auricles - micrognathia - forearm shortening - absence of both thumbs microcephaly	- tracheostomy - bilateral mandibular osteotomy at age 2 - due to bilateral hearing loss, the patient was fitted with a BAHA device - osteotomy of the mandible with placement of internal distractors at age 3 - surgery for removal of the right-sided distractor and removal and implantation of a new right-sided distractor with mandibular osteotomy at age 3 - the patient still remains under the care of the neurology and gastroenterology clinics
4	five	M	Clinical	- micrognathia - low-set auricles - hypotrophy - gothic palate - bilateral hearing loss - abnormally formed thumbs - lack of 12th pair of ribs	- operated atrioventricular septal defect in infancy - secondary palate muscle plication and upper lip and tongue frenulum at age 1 - mandibular body osteoplasty with implantation of distractors at age 5 - removal of distractors in the mandible at age 5
5	newborn	M	Clinical	- hypoplasia of the midface - hypoplasia of the mandible - low-set auricles - hearing loss - cleft soft palate - abnormal structure of the upper and lower limbs	- mandibular osteotomy with placement of distractors at age 1 - removal of distractors at age 1 - pollicization of the thumb of the right hand at age 2 - osteotomy of the mandible with placement of distractors at age 2 - removal of distractors at age 3 - release of ankylosis of the TJM with fat grafting at age 3
6	three	F	Clinical	- hypoplasia of the mandible - low-set auricles - significant bilateral hearing loss - hypoplasia of the upper limbs - cleft palate	- operated abnormalities of the structure of the upper limbs - amputation of the residual underdeveloped thumbs of both hands at age 2
7	two	M	Clinical	- hypoplasia of the mandible - low-set auricles	- tracheostomy - cleft palate surgery at age 6 months - placement of distractors, mandibular osteotomy at age 2 - mandibular body osteoplasty with placement of distractors at age 2 - removal of distractors at age 2 - temporomandibular joint plication at age 2 - removal of right mandibular distractor at age 3
8	two	M	Clinical	- hypoplasia of the mandible - lack of a thumb on both hands - seborrheic dermatitis	- mandibular osteotomy and distraction procedure at age 1 - hearing aid

Legend: M—male; F—female; BAHA—bone-anchored hearing aid.

## Data Availability

Data sharing is not applicable to this article, as no datasets were generated or analyzed in the current study.
